# “Co-construction” in deliberative democracy: lessons from the French Citizens’ Convention for Climate

**DOI:** 10.1057/s41599-022-01212-6

**Published:** 2022-06-22

**Authors:** Louis-Gaëtan Giraudet, Bénédicte Apouey, Hazem Arab, Simon Baeckelandt, Philippe Bégout, Nicolas Berghmans, Nathalie Blanc, Jean-Yves Boulin, Eric Buge, Dimitri Courant, Amy Dahan, Adrien Fabre, Jean-Michel Fourniau, Maxime Gaborit, Laurence Granchamp, Hélène Guillemot, Laurent Jeanpierre, Hélène Landemore, Jean-François Laslier, Antonin Macé, Claire Mellier, Sylvain Mounier, Théophile Pénigaud, Ana Póvoas, Christiane Rafidinarivo, Bernard Reber, Romane Rozencwajg, Philippe Stamenkovic, Selma Tilikete, Solène Tournus

**Affiliations:** 1 Ecole des Ponts ParisTech, Champs-sur-Marne, France; 2 Centre International de Recherche Sur l’environnement et le Développement (CIRED), Nogent-sur-Marne, France; 3 Centre National de la Recherche Scientifique (CNRS), Paris, France; 4 Paris School of Economics, Paris, France; 5 Université Paris 1 Panthéon-Sorbonne, Paris, France; 6 Université de Lille, Lille, France; 7 Centre d’études et de Recherches Administratives, Politiques et Sociales (CERAPS), Lille, France; 8 GIS Démocratie et Participation, La Plaine Sainte-Denis, France; 9 Sciences Po, Paris, France; 10 Institut Pour le Développement Durable et Les Relations Internationales (IDDRI), Paris, France; 11 Laboratoire Dynamiques Sociales et Recomposition des Espaces (LADYSS), Paris, France; 12 Université Paris-Dauphine, Paris, France; 13 Institut de Recherche Interdisciplinaire en Sciences Sociales (IRISSO), Paris, France; 14 Université Panthéon-Assas Paris II, Paris, France; 15 Institut Michel Villey, Paris, France; 16 Université de Lausanne, Institut d’Études Politiques, Lausanne, Switzerland; 17 Université Paris 8, Centre de Recherches Sociologiques et Politiques de Paris (CRESPPA), Paris, France; 18 Harvard University, Ash Center, Cambridge, MA USA; 19 Centre Alexandre Koyré, Aubervilliers, France; 20 ETH Zürich, Zürich, Switzerland; 21 Université Gustave Eiffel, Champs-sur-Marne, France; 22 Centre d’études Européennes et de Politique Comparée (CEE), Paris, France; 23 Université de Strasbourg, Strasbourg, France; 24 Laboratoire Interdisciplinaire en études Culturelles (LinCS), Strasbourg, France; 25 Centre Européen de Sociologie et de Science Politique (CESSP), Paris, France; 26 Department of Political Sciences, Yale University, New Haven, CT USA; 27 Cardiff University, Cardiff, UK; 28 Centre for Climate Change and Social Transformation (CAST), Cardiff, UK; 29 Ecole Normale Supérieure de Lyon, Lyon, France; 30 Triangle. Action, Discours, Pensée Politique et économique, Lyon, France; 31 Rhizome Chôros, Valenciennes, France; 32 Faculté d’Architecture, Université Libre de Bruxelles, Bruxelles, Belgium; 33 Laboratoire Sasha, Bruxelles, Belgium; 34 Université de la Réunion, Saint-Denis, France; 35 Laboratoire de Recherche sur les Espaces Créoles et Francophones (LCF), Paris, France; 36 Centre de Recherches Politiques de Sciences Po (CEVIPOF), Paris, France; 37 Ben-Gurion University of the Negev, Beer-Sheva, Israel; 38 Jacques Loeb Center for the History and Philosophy of Science, Beer-Sheva, Israel; 39 Ecole des Hautes études en Sciences Sociales (EHESS), Paris, France

**Keywords:** Environmental studies, Politics and international relations

## Abstract

Launched in 2019, the French Citizens’ Convention for Climate (CCC) tasked 150 randomly chosen citizens with proposing fair and effective measures to fight climate change. This was to be fulfilled through an “innovative co-construction procedure”, involving some unspecified external input alongside that from the citizens. Did inputs from the steering bodies undermine the citizens’ accountability for the output? Did co-construction help the output resonate with the general public, as is expected from a citizens’ assembly? To answer these questions, we build on our unique experience in observing the CCC proceedings and documenting them with qualitative and quantitative data. We find that the steering bodies’ input, albeit significant, did not impair the citizens’ agency, creativity, and freedom of choice. While succeeding in creating consensus among the citizens who were involved, this co-constructive approach, however, failed to generate significant support among the broader public. These results call for a strengthening of the commitment structure that determines how follow-up on the proposals from a citizens’ assembly should be conducted.

## Introduction

Deliberative mini-publics are gaining traction around the world in addressing a number of complex issues that have proved difficult to solve using the traditional democratic apparatus (Dryzek et al., 2019). The most-studied examples include assemblies on electoral laws in British Columbia and Oregon (Fournier et al., 2011; Warren and Gastil, 2015) and on same-sex marriage and abortion in Ireland (Farrell et al., 2019; Devaney et al., 2020; Courant, 2020). Deliberative mini-publics involve randomly chosen lay citizens who are invited to come together, deliberate and produce policy recommendations. Citizens’ assemblies are a specific form of deliberative mini-public involving a sufficiently large number of participants and lasting long enough for them to submit policy proposals to government executives or elected authorities.

Among the many issues debated in citizens’ assemblies, climate action has come to prominence in the past few years. The French Citizens’ Convention for Climate (CCC) provides the largest-scale experiment to date with climate assemblies—at least measured by the resources it involved (€6.7 million) and the period it spanned (9 months).^1^ The CCC was initiated in 2019 by the President of the Republic in response to what had come to be known as the Gilets Jaunes crisis, a protest movement against the perceived unfairness of government policies—environmental in particular (Nature, 2018; Brancaccio, 2020). It was formally implemented by an engagement letter from the Prime Minister tasking 150 randomly chosen citizens with “defining structuring measures to achieve, in a spirit of social justice, a reduction of greenhouse gas emissions of at least 40% by 2030 compared to 1990”. Nine months later, the selected participants submitted 149 policy proposals, some of which have been included in a new law after intense parliamentary debate.

The CCC had a peculiar feature that makes it a particularly interesting case in relation to studying climate assemblies. In his letter, the Prime Minister referred to the process as an “innovative procedure for co-construction of solutions”. An increasingly popular concept in public administration and management, the notion of co-construction lacks a clear articulation in social sciences. As Jacoby and Ochs (1995) put it, “as a free-standing term, the word co-construction is quite elliptical, implying some nonspecified joint activity of creation, deliberately leaving one in the dark as to who (or what) might be acting in concert and what exactly is being jointly created”. In the context of the CCC, the concept was brought up as a means to cope with the social justice imperative, but the government did not provide further guidance on how to implement it.

Taken in the broadest sense that something is being jointly created, co-construction shares the same goal as a citizens’ assembly—increasing democratic quality. Yet the broad involvement of external actors alongside the citizens may appear at odds with the core principle of a citizens’ assembly: that it relies on a selected few. This raises two important questions. First, if non-citizen, professional bodies (say, experts, organizers, facilitators) are to play an active role in a citizens’ assembly, as a co-constructive approach would require, who then is responsible for the final output? Second, can co-construction be envisaged without the broader public, which by design is excluded from a citizens’ assembly?

Taking France as a case study, we examine these questions in three steps. To begin with, we provide a synthetic account of the CCC proceedings and its subsequent developments. We then address the first question, asking whether and how external input affected the citizens’ agency, creativity and freedom of choice. In doing so, we examine the interactions that took place between the citizens and the steering bodies at three key stages of the deliberation process—agenda-setting, drawing up of proposals and decision-making. We find that the steering bodies, in particular the governance committee, exerted significant framing effects on the citizens’ deliberation. Yet the framework remained flexible enough to preserve the citizen’s independence, which for instance allowed them to take the carbon tax issue out of the agenda. Overall, the citizens selected ideas that were subsequently refined into detailed proposals with the help of the legal and technical advisory groups. We then turn to the second question and examine whether and how the citizens’ work resonated with the broader public. We find that the interactions between the citizens and the broader public were characterized by mutual skepticism. The CCC developed as an autonomous body, somewhat isolating itself from the broader public, seen as a potential impediment in their quest to produce a coherent and consistent set of measures. In the same vein, the citizens turned down the opportunity of submitting their proposals to referendum. Altogether, the co-constructive approach to deliberative democracy that prevailed throughout the CCC did not undermine the citizens’ responsibility over the final output but failed to generate resonance with the broader public. The article closes with a call for a clear commitment structure giving the citizens more visibility ex ante on how their proposals are to be followed up on ex post.

Our analysis builds on our unique experience as observers of the CCC. We were part of a group of accredited social science researchers who worked collaboratively to document and analyze the CCC. In this paper, we exploit several different sources of research material: the qualitative observations each of us collected and shared with the group; quantitative data from internal and external surveys; and voting data generated during the proceedings.

The remainder of the paper is organized as follows. Section “Background” provides some theoretical background and explains the research questions. Section “Methods” describes the materials and methods used. Section “The CCC proceedings: an overview” describes the CCC proceedings, detailing its structure, process, and early outcomes. Section “Interactions between the citizens and the steering bodies” examines the interactions between the citizens and the steering bodies. Section “Interactions between the citizens and the general public” examines the interactions between the citizens and the broader public. Section “Taking stock: Researching the CCC” provides feedback on our research experience. Section “Conclusion” concludes with some policy recommendations.

## Background

Our analysis lies at the intersection of two research themes, namely citizens’ assemblies and co-construction. Here, we review the associated literature and highlight the connections between the two themes in relation to climate action.

### Deliberative democracy and climate action

From a political procedural perspective, citizens’ assemblies both complement and feed into representative democracy in an attempt to increase the quality of deliberation, thereby making policy fairer and more effective. To achieve this, citizens’ assemblies are intended to foster authentic, inclusive and consequential deliberation (Dryzek, 2009). Deliberation is a form of structured exchange of arguments, information and stories that can, when conducted properly, produce and harness collective intelligence. Together with rhetoric—the main vehicle in conventional democracy (Chambers, 2009)—it contributes to reflecting the will of the many. Random selection of participants is expected to make deliberation in citizens’ assemblies both more authentic and more inclusive, enabling all types of citizens—chiefly including those who do not normally participate in popular votes (Neblo et al., 2010)—to bring and share their first-hand experience of practical yet complex problems. The citizens’ assembly in turn serves as a “recommending force” or “body of trustees” to the broader public (Mansbridge et al., 2012; Warren and Gastil, 2015). There is growing evidence from the United States (Gastil et al., 2016; Ingham and Levin, 2018), Canada (Boulianne, 2018), and Ireland (Suiter et al., 2020) that mini-publics are indeed effective at generating support among the broader public. Such endorsement is thought to rely on different processes, including trust—the broader public perceiving the mini-public as representative, hence trustworthy—and heuristics—assuming some division of cognitive labor is inevitable in democratic decision-making, the broader public can thus rely on “information cues” produced by the mini-public (MacKenzie and Warren, 2012).

The degree to which deliberation in citizens’ assemblies should be consequential is less consensual. While most theorists agree that participants who have devoted significant time and effort to such a process should at least “have a say” in its outcomes (Chambers, 2009; Goodin and Dryzek, 2006; Caluwaerts and Reuchamps, 2015), arguments differ as to the form this should take. On the positive side, some advocate that citizens’ assemblies should be systematically embedded into public decision-making (Fishkin, 2018). On the skeptical side, in contrast, others view citizens’ assemblies as “democratic shortcuts” that, if overly relied on, illegitimately bypass the will of the broader public (Lafont, 2015). In between, some suggest that pairing citizens’ assemblies and a referendum (perhaps iteratively) can provide the best of both worlds (Setälä, 2011, 2017; Landemore, 2018; Parkinson, 2020). In practice, with increasing empirical evidence gathered from nearly 200 experiences with deliberative mini-publics (Smith, 2009; Paulis et al., 2020; Jacquet and van der Does, 2020), the device has proved mostly inconsequential so far, with politicians cherry-picking from its conclusions. One important exception is Ireland, where citizens’ assemblies were followed up with three referenda, two of which resulted in the same outcome as that recommended by the mini-public (Suiter and Reidy, 2020).

Climate action has recently become an important focus in citizens’ assemblies. From a normative perspective, it has been noted that deliberative democracy and climate change are closely connected. Climate change is a complex, urgent, and, by and large, intangible problem. Its effects are felt with distance across both space and time, thereby lacking salience—a complication representative democracy has fallen short of addressing (Niemeyer, 2013). The inclusiveness inherent in deliberative democracy is thought to be better fitted to overcome this limitation (Dryzek and Stevenson, 2011; Burnell, 2012; Baber and Bartlett, 2021). Perhaps most crucially, climate assemblies uniquely question the role experts should play in deliberative democracy. The sheer scale of the problem at hand and its highly technical nature make expert input essential in the citizens’ deliberation. Specifically, expert input can serve as a “selectively convergent” base for judgment and thus contribute to building trust among citizens (Warren and Gastil, 2015; Hendriks, 2006). Reaping these benefits however requires a great deal of transparency about experts’ interests and careful oversight of their interaction with the citizens.

### Co-construction of public policy

Co-construction is an increasingly popular concept in management, public administration, science and technology studies, and sustainability science (Miller and Wyborn, 2020). It is somewhat broader, yet much less well articulated, than deliberative democracy. Accordingly, the related literature is much scarcer. To complicate matters further, co-construction is often used interchangeably with the related concepts of co-creation and co-production (Vaillancourt, 2009; Brandsen et al., 2018).^2^ In what follows, we focus on the co-construction terminology while largely building upon the common basis for the three concepts.^3^

Broadly speaking, co-construction consists of involving citizens alongside state representatives in the making of policy, with the goal of increasing the democratic level of public service delivery (Brandsen et al., 2018; Brandsen and Honingh, 2018). The citizens are involved as (groups of) individuals, as opposed to organizations. They receive professional support to help them increase their competency (Verschuere et al., 2018).^4^ Importantly, the parties involved share responsibility for the outcome. This specific form of accountability is expected to increase citizens’ trust in public institutions, and trust in society in general (Fledderus, 2018).

### A co-constructive citizens’ assembly?

Thus defined, citizens’ assemblies and co-construction share the same goal of increasing democratic quality. They nonetheless differ in two important respects, which provide the research questions leading to our analysis. First, a sort of primacy of the citizens prevails in citizens’ assemblies. External input (from experts in particular), while allowed, is limited to that which is strictly necessary. In contrast, under co-construction, the outcome might be of greater consequence, but responsibility for it is somewhat shared—or diluted, as some warn (Steen et al., 2018)—between the citizens and the state. Who then can be held responsible for the outcome of a co-constructive citizens’ assembly, as is asked of the CCC? Second, whereas co-construction does not specify how citizens get involved, citizens’ assemblies are comprehensively structured around a sortition process. On the one hand, this helps address a common criticism that co-construction is not necessarily inclusive and representative of the diversity of citizens (Brandsen et al., 2018; Verschuere et al., 2018; Steen et al., 2018). On the other hand, it draws a clear line between participants and non-participants, whereas the line is more blurred with co-construction. What role then can non-participants (i.e., the public at large) play in a co-constructive citizens’ assembly?

Answering these questions requires close, comprehensive scrutiny of the proceedings and their broader context. In the next section, we describe the research protocol we implemented to achieve this.

## Materials and methods

Through an open call, the CCC’s governance committee invited up to 40 researchers to closely follow the process. This led to the formation of a group of 30 social scientists from various disciplines—political science, economics, sociology, philosophy, geography, law—actively working together to collect qualitative and quantitative data during the process.

### Observation

The governance committee and the facilitators granted us wide access to plenary and group discussions. Identified with a “Researcher” tag, we were allowed to observe the citizens’ interactions, take notes and make audio recordings of their conversations. We were also allowed (with camera and audio turned off) to follow the webinars that took place between the face-to-face sessions. In return, we adhered to a charter in which each of us committed not to interfere with the process. This implied observing debates at a reasonable distance, engaging as little as possible with participants and organizers, refraining from publicly expressing personal views on the CCC during the process and from communicating preliminary research results (see Supplementary Appendix A).

Our group set out to cover the whole process as systematically as possible. We formed small teams of one to five researchers, grouped either by discipline, thematic interest or home institution. We then scheduled individual attendance so as to meet two objectives: have all events covered at the whole-group level, including both the plenary sessions and the parallel group discussions; and maintain a permanent presence of each team throughout the process. These requirements were demanding, considering the short notice (3 weeks) between the circulation of the call and the beginning of the CCC, the planning of the sessions on weekends and the many disruptions that occurred in the schedule (see section ‘Process’). They were nonetheless to a large extent fulfilled, with attendance never falling below 20 researchers, about two-thirds of us being able to follow at least 50% of the proceedings, and five of us even following 100% of it. At the beginning of each session, our plenary group would meet in person to discuss the protocol and agree on the allocation of teams and researchers across parallel sessions. In between sessions, we would meet remotely to share observations and discuss preliminary findings. A follow-up discussion was organized in July 2020 and the different teams shared their observations and findings in a public workshop held remotely on November 17–18 2020. The observations reported here are those that proved most convergent among us.

### Internal survey

We prepared questionnaires to survey the citizens’ values, their attitudes towards climate change and their feelings and views about the Convention. We initially planned to survey the citizens both at the beginning and at the end of each session. In an attempt to capture changes, the questionnaires included repeated questions alongside more session-specific questions. Unfortunately, while the response rate was high in the first two sessions, it sharply declined thereafter, before coming back to average in Session 7. As a result, some questionnaires are challenging to exploit. In this paper, we provide some results for the sessions with the highest response rates, namely Sessions 1, 2, and 7. In Session 1, we surveyed general values, perceptions of climate change and attitudes towards selected climate policies (*N* = 111 to 136). In Session 7 (*N* = 63 to 65), we surveyed perceptions of the CCC and of the citizens’ experience as participants. Questionnaires and descriptive statistics can be found here: https://www.participation-et-democratie.fr/donnees-de-recherche-sur-la-convention-citoyenne-pour-le-climat.

### External survey

In parallel, we surveyed a representative sample of the French population to assess the CCC’s representativeness in terms of values and political opinions, track the evolution of the citizens’ opinions, and document perceptions of the CCC. To facilitate comparison, we asked the same questions in both the external and internal surveys. Before the start of the Convention, we surveyed the external sample about their socio-demographic characteristics, perceptions of climate change, attitudes towards climate policies, general values, and perceptions of the CCC. The survey was conducted in two waves, each administrating the same questionnaire to a specific sample of one thousand people each. The two samples were representative of the French population based on the same socio-demographic characteristics as those considered in the CCC (see Table 1). The first wave was administered between April 22 and May 11, 2020, before the CCC’s proposals were made public on June 21. The second wave was administered between October 19 and November 3, 2020, after the CCC’s proposals had started being discussed in the general debate. The original questionnaire can be found online at http://preferences-pol.fr/doc_q.php#_c and the results are analyzed in detail in Fabre et al. (2021).

**Table 1:** Composition of the CCC and of the respondents to the external survey (first and second waves).

		French population	Participants in Session 1	Participants in Session 7	External survey, W1	External survey, W2
		*N* = 67 million	*N* = 159	*N* = 160	*N* = 1003	*N* = 1003
Gender	Female	47.8%	49.1%	48.1%	44.6%	48.5%
	Male	52.2%	50.9%	51.9%	55.4%	51.5%
Age	16-17	3.0%	3.1%	4.4%	0.0%	0.0%
	18–24	10.6%	9.4%	8.8%	11.7%	10.6%
	25–34	15.3%	16.4%	15.0%	14.5%	15.3%
	35–49	25.3%	21.4%	21.9%	20.2%	19.2%
	50–64	24.1%	30.2%	31.9%	26.0%	26.3%
	Over 65	21.8%	19.5%	18.1%	27.6%	28.6%
Socio-economic group	Farmers	0.9%	1.3%	0.6%	0.9%	0.8%
	Small entrepreneurs	3.5%	3.8%	4.4%	4.2%	3.5%
	Managers and professionals	9.2%	13.8%	13.8%	9.8%	9.1%
	Technicians and associated professional employees	14.3%	17.0%	15.0%	10.4%	14.1%
	Clerks and skilled service employees	16.8%	12.6%	14.4%	17.9%	18.9%
	Industrial skilled employees	13.3%	8.2%	9.4%	13.7%	14.6%
	Retired	27.2%	27.0%	26.3%	28.1%	27.9%
	Other non-employed	14.9%	16.4%	16.3%	15.1%	11.2%
Highest qualification	No diploma	27.6%	23.9%	25.0%	18.4%	18.7%
	Pre-baccalaureate	22.0%	17.0%	18.8%	29.5%	27.7%
	Baccalaureate	15.1%	18.9%	17.5%	16.2%	17.5%
	Post-baccalaureate	25.9%	28.3%	26.3%	30.4%	30.1%
	Currently student	9.4%	12.0%	12.5%	5.5%	5.9%
Settlement	Urban	59.0%	61.0%	62.5%	NA	NA
	Sub-urban	24.0%	21.4%	18.8%	NA	NA
	Rural	17.0%	13.8%	15.6%	NA	NA
	Other	0.0%	3.8%	3.1%	NA	NA
Location	Auvergne-Rhône-Alpes	11.8%	10.1%	12.5%	NA	NA
	Bourgogne-Franche-Comté	4.4%	1.3%	1.3%	NA	NA
	Bretagne	5.0%	5.0%	5.0%	NA	NA
	Centre-Val de Loire	3.9%	4.4%	3.8%	NA	NA
	Corse	0.5%	0.6%	0.6%	NA	NA
	Grand Est	8.6%	6.3%	7.5%	NA	NA
	Hauts-de-France	9.0%	12.0%	11.9%	NA	NA
	Île-de-France	17.9%	25.2%	23.1%	NA	NA
	Normandie	5.1%	2.5%	1.3%	NA	NA
	Nouvelle-Aquitaine	9.1%	8.2%	8.8%	NA	NA
	Occitanie	8.8%	7.6%	6.3%	NA	NA
	Pays de la Loire	5.5%	5.7%	5.6%	NA	NA
	Provence-Alpes-Côte d’Azur	7.7%	7.6%	9.4%	NA	NA
	Guadeloupe	0.6%	1.3%	1.3%	NA	NA
	Martinique	0.6%	0.6%	0.0%	NA	NA
	Guyane	0.3%	0.6%	0.6%	NA	NA
	La Réunion	1.2%	1.3%	1.3%	NA	NA

Note: Settlement and location were not coded in the external survey in the same way as in the CCC, hence the NAs.

### Official material

We had access to an online internal platform that was set up for the citizens to aid circulation of information and enable collaboration. In addition to our own survey data, we exploited the quantitative data generated during the voting sessions (CCC, 2020, 2021).

## The CCC proceedings: an overview

After briefly introducing the context that brought about the CCC, we describe its proceedings using the common structure-process-outcome framework (Goold et al., 2012)^5^ and discuss the extent to which the citizens’ proposals have been followed up on.

### Context

In November 2018, France experienced a major political crisis. In response to a set of government measures deemed unfair to the poor—a planned increase in the carbon tax, a reduction of speed limits from 90 to 80 km/h mainly applying in rural areas and tax cuts benefiting the rich—protesters started gathering every Saturday and occupying roundabouts on a daily basis (Nature, 2018; Brancaccio, 2020). What came to be known as the Gilets Jaunes (or Yellow Vests) movement made the headlines of French political life for nearly 6 months, with aftershocks still being felt. Among other responses, the government organized what was termed the “Grand National Debate,” a forum including elements of participatory and deliberative democracy, in particular 10,000 local debates and 18 “regional citizen conferences,” each inviting 70 to 100 randomly selected citizens to deliberate for a day and a half. In closing the Grand National Debate in April 2019, President Macron took a step further. Responding to a call from a group of activists called Gilets Citoyens, he announced the creation of a dedicated citizens’ assembly on climate—the CCC (see announcement in Supplementary Appendix B). The President made the commitment that the measures submitted by the citizens’ assembly would be brought “without filter” to the appropriate level: referendum, government or parliamentary action. In thus committing to take the citizens’ proposals undiluted or unchanged, the President asked in return that the citizens produce readily implementable bills.

The CCC was formally initiated in July 2019 by an engagement letter from the Prime Minister (reproduced in Supplementary Appendix C) inviting participants to “define structuring measures to manage, in a spirit of social justice, to cut France’s greenhouse gas (GHG) emissions by at least 40% by 2030 compared to 1990”.^6^ The letter was addressed to the head of the Economic, Social and Environmental Council (CESE), to whom the organization of the CCC was delegated.^7^ The 40% target corresponded at the time to France’s intended nationally determined contribution submitted in compliance with the Paris Agreement.^8^ The emphasis on social justice was meant to overcome the pitfalls that had created the Gilets Jaunes crisis.

### Structure

At the Prime Minister’s request, two committees were set up to organize and scrutinize the work of the citizens—a governance committee and a guarantors’ college. Bringing together representatives from various organizations, including members from the Gilets Citoyens group, the governance committee was tasked with setting the agenda, defining procedures, supervising the process and providing legal and technical support (cf. Supplementary Appendix C). The Prime Minister nominated two think tank representatives, Thierry Pech and Laurence Tubiana, as co-chairs of the governance committee. They in turn appointed 13 fellows from various organizations (think tanks, unions, businesses, academia). The governance committee further included two seats for citizens whose occupants rotated between sessions. The governance committee further appointed two spin-off groups: a technical advisory group of 19 experts with different backgrounds—policy, business, economics, sociology—and a legal advisory group of six experts. The latter was to provide support for the legal transcription of the proposals, a prerequisite for them being followed up on “without filter” by the President of the Republic. To our knowledge, such legal support has no precedent in other citizens’ assemblies. The guarantors’ college had three members. Their role was to oversee the impartiality and sincerity of the process. They produced a report at the end of each session.

The participating citizens were selected in August and September 2019. From an initial pool of 300,000 randomly generated phone numbers, contact was made with 11,400 people to survey their socio-economic characteristics and their willingness to participate. Among the positive respondents, 190 were selected in an effort to fill quotas based on age, gender, education level, geographical origin, settlement (urban versus rural) and type of job (if any) (see Table 1). Importantly, unlike in other citizens’ assemblies (e.g., CAUK), attitudes towards climate change were not part of the selection criteria. Of the 190 candidate participants, 178 were effectively summoned, of whom 104 effectively participated in all sessions, 56 participated in some but not all sessions, 10 never showed up and 8 dropped out along the way. The number of citizens who were ultimately considered official participants is 159.

The question naturally arises as to the degree to which the selected participants are representative of the general population based on a broader set of criteria. As it turns out, the views expressed in the internal surveys on general issues such as education and political leanings are a fairly good match for those expressed in the external surveys (Fabre et al., 2021). The key differences are a more pronounced concern for the environment and a greater confidence in others among CCC participants.^9^ Since participation was voluntary and selection ignored attitudes towards climate change, such biases could arguably not be avoided.

Alongside the citizens, the governance committee, its spin-off committees and the guarantors’ college, a consortium of facilitators was procured to fulfill the role of leading the debates. A budget of €4.5 million was initially planned to organize the CCC, most of which was dedicated to logistics, compensation for the citizens—each participant received a daily allowance of €84 (hence €1462 over the whole course of events), plus specific benefits for child care and lost income—and the facilitators’ service fees. Total costs eventually reached €6.7 million.

### Process

The CCC was initially scheduled to span six three-day sessions (Fridays through Sundays), from October 2019 to early February 2020. Two major events disrupted these plans. First, protests against a pension reform led to France’s longest strike in decades. Public transport was nearly shut down from early December 2019 to mid-January 2020, thus delaying Session 4. By that time, the citizens had been granted a seventh session at their request. Second, soon after Session 6, lockdown was ordered to fight the COVID-19 outbreak. After two interim sessions were held remotely during the lockdown period, the final session (#7) was held at CESE with social distancing measures on June 19–21—4 months later than initially planned.

The CCC sessions combined plenary gatherings and parallel gatherings held in smaller thematic groups. The thematic groups were defined by the governance committee so as to cover five relevant sources of GHG emissions: housing (*Se loger*), production and labor (*Produire et travailler*), transport (*Se déplacer*), food (*Se nourrir*), and consumption (*Consommer*). The citizens were randomly assigned to a thematic group. Within this governance committee-imposed framework, they found room to adjust the agenda, as we will see in Section “Interactions between the citizens and the steering bodies”.

The CCC had several stages (See Table 2). In Session 1, citizens heard from climate scientists and were introduced to the aims and scope. In a second stage spanning Sessions 2 to 6, they interrogated experts, debated and elaborated policy proposals. Under the guidance of facilitators, they would alternate hearings with experts and round-table discussions, in either plenary or thematic gatherings. Between sessions, the citizens’ proposals would be compiled by the facilitators, assessed by the technical advisors and reformulated by the legal advisors in legal terms. At the next session, the citizens would start with their reworked proposals and engage in another round of adjustment. In Session 6, each group presented their work in plenary gatherings to get feedback from other groups. After Session 6, all citizens were invited to suggest amendments to the final proposals and to vote for or against those amendments that were supported by at least 20 citizens. This resulted in 150 measures submitted by the thematic groups to the Convention as a whole.

**Table 2:** Timeline of the CCC.

Session 1	Session 2	Session 3	Session 4	Session 5	Session 6	Session 7	Session 8
4–6 October 2019	25–27 October 2019	15–17 November 2019	10–12 January 2020	7–9 February 2020	6–8 March 2020	19–21 June 2020	26–28 February 2021
**INTRODUCTION**	**THEMATIC OVERVIEW**	**INCEPTION**	**CONSOLIDATION**	**FINALIZATION**	**VALIDATION**	**CLOSURE**	**FEEDBACK**
• Introductions • Objectives • Introduction to climate change	• State of the art • Controversies • Solutions	• Preliminary solutions • Preliminary assessment of their contribution	• Separation of proposals and recommendations • Identification of cross-cutting issues	• Engagement with policy-makers • Debate • Validation of report outline	• Thematic work presented in plenary gatherings • Writing of the report	• Final votes • Submission of the final report	• Feedback from experts on follow-up • Votes on follow-up appraisal

**Fig. 1:**
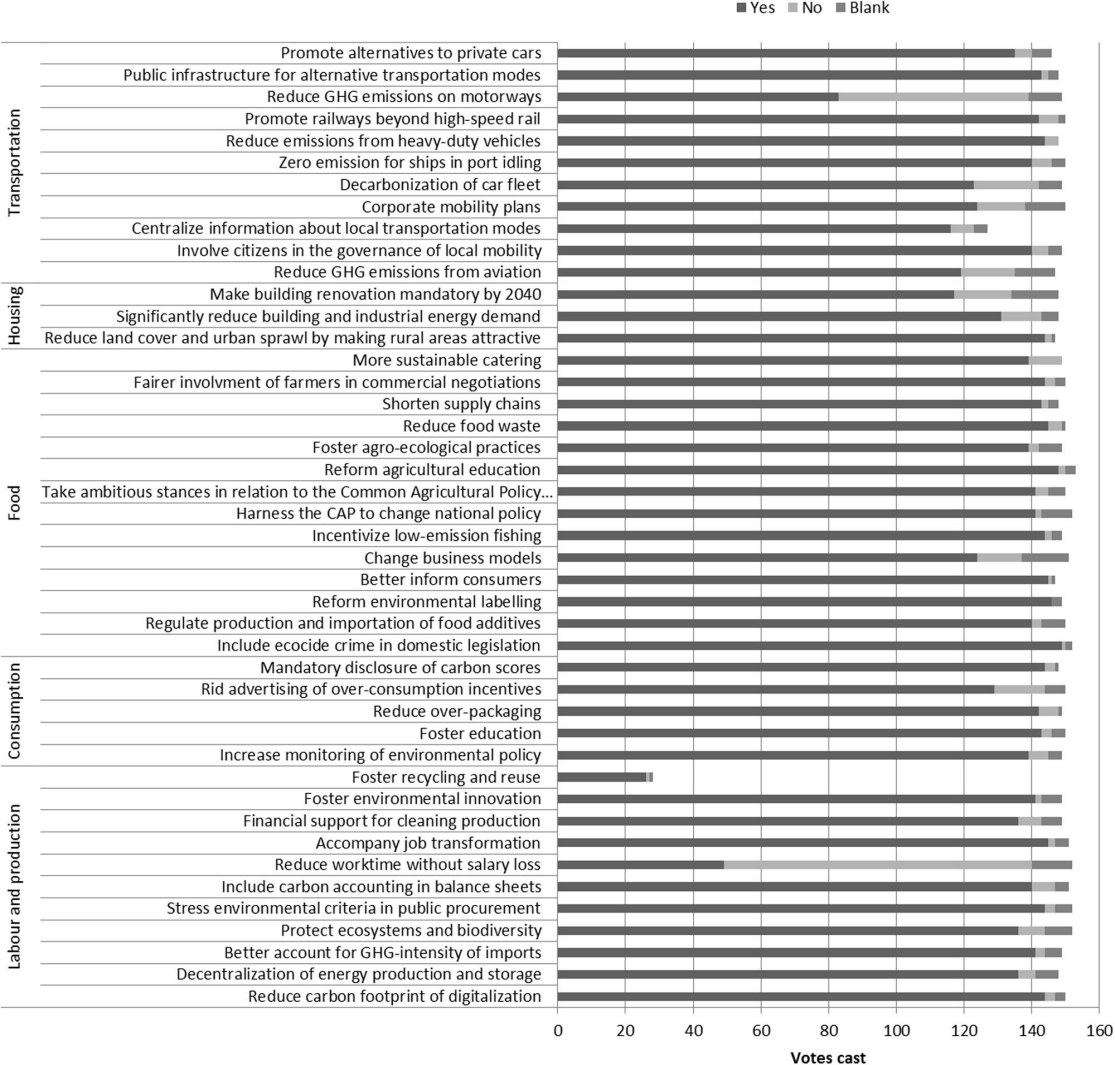
Support for the 44 blocks of measures. The histograms indicate the votes cast for each block of measures.

In the third and final stage of the process (Session 7), the full body of citizens participated in a series of votes. In a first voting phase, they were asked whether they approved of each of the measures, grouped into 44 blocks of 1 to 13 coherent proposals (see Fig. 1). In a second phase, they voted on whether a subset of technical measures deemed legally fit should be submitted to a referendum, a provision that was included in the Prime Minister’s engagement letter (see Supplementary Appendix C). All votes were held on a simple majority basis. Voting was supervised by the guarantors’ college.

The CCC was relatively openly publicized. The media were given extensive access to the CCC’s gatherings and proceedings. Participating citizens’ surnames were kept anonymous by default but they were free to go public on social or traditional media. They were also encouraged by the organizers to reach out to their local community between sessions and meet with various stakeholders such as businesses, unions, members of parliament and local elected representatives. While some plenary gatherings were broadcast on YouTube, the governance committee decided that group deliberations and the drafting of proposals would be kept confidential from Session 6 onwards in an effort to prevent external influences from impinging on the content of the measures.

At different points in the process, plenary meetings were organized between the citizens and the highest executives of the French State—the Minister for the Ecological and Inclusive Transition (Session 1), the Prime Minister (Session 1) and the President of the Republic (Session 4)—to clarify their mutual expectations.

### Outcome

The first voting phase resulted in all blocks of measures but one being approved. The one rejected measure was a proposal to reduce working time from 35 to 28 h a week, which many citizens deemed “unrealistic” in the deliberation. Other blocks of measures received between 85 and 100% approval, except for one block—proposing a lowering of speed limits from 130 to 110 km/h on motorways—which was only approved by 60% of the votes cast. Altogether, 149 measures from 43 blocks were approved (see scores in Fig. 1). In the second voting phase, participants approved two constitutional reforms—rephrasing the Preamble and Article 1 of the Constitution—and the proposal that recognition of the crime of ecocide be put to a referendum. Meanwhile, a majority voted against putting those technical measures deemed legally fit to a referendum.

To cover the cost of these measures, 78 financing schemes were proposed, of which 75 received majority support in a final vote (turn-out: 105). Support was above 70% for a few landmark measures, such as increasing public debt, implementing new taxes (on wealth, advertising, unhealthy food, carbon border adjustment) and introducing increasing progressivity into existing tax schemes (e.g., increasing income tax for households whose annual income exceeds €250,000).

Most of the proposals were national in scope. However, other relevant dimensions of the problem were not ignored. On the one hand, a number of measures concerning agriculture, land-use, and public transportation were differentiated at the local level to take into account stronger vulnerability to climate change, in particular in overseas territories. On the other hand, the citizens made recommendations for France’s foreign policy relating to climate affairs, in particular by recommending that negotiations over trade agreements (in particular the Comprehensive Economic and Trade Agreement with Canada) be paused so environmental conditions could be added.

Whether the measures were consistent with the target of reducing France’s GHG emissions by 40% by 2030 had not been comprehensively assessed at the time of voting. Only rough estimates of the impact of each measure were provided to the participants (on a low-medium-high scale), with no assessment of their combined impact (see Table 3). These estimates were put together by the technical advisory group and shared with the citizens only days before the final vote was held. Likewise, the degree to which the measures met the social justice imperative was not systematically assessed. Yet most of the proposals provided extra-benefits or exemptions to low-income households. In contrast to GHG emissions, policy costs were assessed for a few measures deemed to have the most impact. The technical advisory group estimated the annual cost of the 149 measures to be in the region of €6 billion, including €1 billion each for the obligation to retrofit the least energy efficient dwellings, tightening of fuel efficiency standards, development of rail transport, and restrictions on air travel (I4CE, 2020).

**Table 3:** Strength of the impact of the blocks of measures proposed in each sector, as assessed by the technical advisory group.

	High	High to medium	Medium	Medium to low	Low	N/A
Transportation	• Decarbonization of car fleet	• Promote alternatives to private cars • Public infrastructure for alternative transportation modes • Promote railways beyond high-speed rail • Reduce emissions from heavy-duty vehicles	• Reduce GHG emissions on motorways • Reduce GHG emissions from aviation		• Zero emissions for ships in port idling • Centralize information about local transportation modes • Involve citizens in the governance of local mobility	• Corporate mobility plans
Housing	• Make building renovation mandatory by 2040 • Reduce land cover and urban sprawl by making rural areas attractive		• Significantly reduce building and industrial energy demand			
Food	• Foster agro-ecological practices		• More sustainable catering • Fairer involvement of farmers in commercial negotiations • Shorter supply chains • Reduced food waste • Reform of agricultural education • Ambitious stances in relation to the Common Agricultural Policy (CAP) • Harness the CAP to change domestic policy • Change business models • Better inform consumers • Include ecocide crime in domestic legislation			
Consumption			• Mandatory disclosure of carbon scores • Rid advertising of over-consumption incentives		• Reduce over-packaging	• Foster education • Increase monitoring of environmental policy
Labor and production	• Better account for GHG-intensity of imports		• Financial support for cleaning production • Include carbon accounting in balance sheets • Stress environmental criteria in public procurement • Decentralization of energy production and storage	• Foster environmental innovation	• Foster recycling and reuse • Accompany job transformation • Protect ecosystems and biodiversity • Reduce carbon footprint and digitalization	• Reduce worktime without salary loss [not retained]

### Follow-up

A week after the final session, President Macron hosted a public meeting with the citizens at the Elysée Palace.^10^ He committed to supporting 146 of the 149 proposals, invoking three “veto cards” (*jokers*) to reject the following measures: changing the Preamble of the Constitution, arguing it threatened to place the protection of Nature above all liberties; imposing a 4% tax on corporate dividends to finance climate action, arguing it would be too damaging for France’s competitiveness; and reducing speed limits on motorways, arguing he had made a similar mistake in the past, thus referring to one of the measures that sparked the Gilets Jaunes movement.^11^

In the autumn of 2020, the government started taking forward the Conventions’ proposals, which included implementing decrees, passing new bills and organizing the only referendum that had not been vetoed by the President—changing Article 1 of the Constitution.^12^ The government drafted an all-encompassing bill reworking the CCC’s proposal. Several meetings were organized to share this work with the members of the CCC, one meeting even involving the President of the Republic on December 14th, 2020. The draft bill was submitted by the government on February 10th, 2021. It was accompanied by an impact assessment study estimating that, if enacted, the measures would lead to between half and two-thirds of the target being reached (Assemblée Nationale, 2021). The High Council on Climate later pointed to limitations in this assessment, suggesting that the true impact was even lower (HCC, 2021).

Later in February 2021, the citizens were summoned for an eighth and final three-day session to evaluate the government’s response to their proposals, as the Prime Minister’s letter had recommended (see Supplementary Appendix C). They were first given feedback from the CCC’s technical and legal advisory groups on whether and how accurately their measures had been followed up on. In a series of 58 votes, they were then asked to appraise the whole process and the government’s follow-up (on a 0–10 scale). As Table 4 illustrates, they judged the outcome severely (Q1 and Q2), with scores in the 2–3 range. In Q1 as well as in many other measure-by-measure votes (CCC, 2021), a block of about 20 ballots voted zero, whatever the degree of follow-up that had been assessed by the advisory groups, suggesting that some citizens voted strategically to express strong disapproval. On the other hand, the citizens expressed positive feelings about citizens’ assemblies in general (Q3 and Q4).

**Table 4:** Voting results for the four general questions posed in Session 8.

		Registered	Null	Blank	Counted	Average grade	Median grade	Standard error
Q1	What is your feeling about the government’s follow-up on the Convention’s proposals?	123	25	2	96	3.3	3	2.6
Q2	To what extent does the government’s follow-up on the Convention’s proposals enable the 40% GHG emissions reduction target to be achieved in a spirit of social justice?	123	25	6	92	2.5	2	2.4
Q3	To what extent did the Convention contribute to climate change mitigation in France?	123	24	15	84	6.0	7	2.7
Q4	In your opinion, to what extent can citizens’ assemblies improve democratic life in our country?	123	23	2	98	7.6	8	2.3

After the eighth session, the bill was debated in Parliament for several weeks, generating over 7000 amendment proposals in the National Assembly—a high number by that body’s standards. The Senate and the National Assembly finally agreed on a law on July 20th.^13^ Meanwhile, the President did not obtain the support needed from Congress^14^ to organize the referendum to change the Constitution. As of today, many decrees remain to be passed for the law to come into force.

Over the course of the CCC, around a dozen citizens rose to prominence in the public arena, owing to their activity on social media or repeated appearance in the traditional media. One citizen later published a book about his experience (Fraty, 2021). At least six citizens have joined some of the leading political parties and run for elections. Some of them are now in office at different levels—regional, municipal and European. Towards the end of the process, some citizens created a non-profit organization and set out to monitor the long-term impact of the CCC output (Les 150. L’association des citoyens de la Convention Climat, https://www.les150.fr/).

## Interactions between the citizens and the steering bodies

The CCC involved a number of steering bodies alongside the citizens, including the governance committee, the guarantors’ college, the technical and legal advisory groups and the facilitators. To our knowledge, such a plethora of supervisory bodies is unparalleled in other citizens’ and climate assemblies. We examine here the extent to which the interactions between the citizens and these bodies affected responsibility for the output. We focus on three key stages of the process—agenda-setting, drawing up of proposals and decision-making—and consider several dimensions of responsibility—agency, creativity and freedom of choice.

### Agenda-setting

The Prime Minister gave the task of setting the agenda to the governance committee. Perhaps its most important intervention in this regard was in framing the five thematic groups around energy demand, leaving energy supply issues—and in particular the role nuclear power should play in electricity generation^15^—largely unaddressed. Yet agenda-setting was not unilateral and the citizens exerted agency to adjust it on two important occasions.

**Fig. 2:**
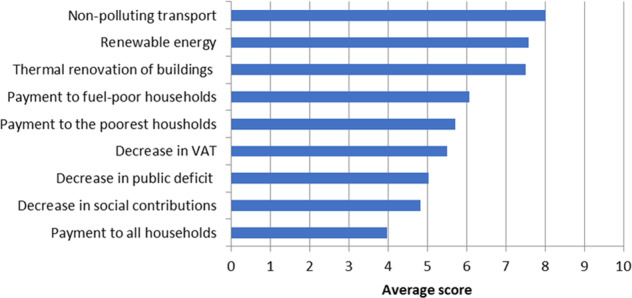
Preferences regarding carbon tax-revenue recycling. Surveyed in Session 2, between 111 and 118 respondents, depending on the options. The question asked was: “To what extent [on a 0–10 scale] would you accept an increase in the carbon tax if the revenue were used to…”.

The first adjustment occurred when some citizens opposed adding the carbon tax to the agenda. The issue had been sensitive from the beginning, as the carbon tax was widely regarded as a tipping point into the Gilets Jaunes crisis. Important expectations therefore followed as to what the CCC would do with respect to the carbon tax—keep its rate frozen or resume the planned increase. When surveyed in Session 1, 72 citizens out of 136 respondents (hence at least 45% of the 159 participants) supported an increase in the carbon tax to limit GHG emissions. At the beginning of Session 2, support was still significant, with 111 citizens expressing high approval rates for a tax increase, provided its revenue were used to finance mitigation measures (Fig. 2). Then in the course of Session 2, during a plenary session in which economic experts were invited to discuss the pros and cons of the carbon tax, a few citizens vehemently interrupted the discussion, arguing they were not there to make up for the government’s shortcomings on the carbon tax. After this dramatic episode, the issue was never raised again. Meanwhile, support for the carbon tax dropped. When invited to vote in Session 7 on whether they supported a 5-year moratorium before discussing any increase in the carbon tax rate, 30% were strongly positive, 27% were positive, 8% were negative and 20% were strongly negative (turn-out: 105).

Another adjustment occurred in the way cross-cutting issues were handled. The governance committee had initially planned to create a cross-cutting issues group on the carbon tax. After the citizens discarded the issue, the focus of the cross-cutting issues group shifted to financing issues and constitutional changes. The so-called “squad” group (*l’escouade*) was formed in Session 3, including both volunteers from among the citizens and others who were randomly chosen, all leaving their home thematic group when deliberating on cross-cutting issues. This raised two major criticisms from non-members, many of them arguing that cross-cutting issues could not formally be separated from specific ones, and that having members from their home group—often the most actively engaged ones—temporarily leave to join the cross-cutting issues group would weaken the former. To relieve tension, the governance committee terminated the cross-cutting issues group at the end of Session 4 and scheduled cross-cutting issues to be discussed in plenary sessions.

### Drawing up of proposals

The CCC asked the citizens to formulate policy proposals, not just to approve of measures from a pre-defined list, as has been the case in many other assemblies (OECD, 2020). Furthermore, the proposed measures had to be readily implementable—a counterpart to the President’s “no filter” commitment. These requirements strengthened the need for expert input already inherent in deliberations over climate policy, a particularly wide-ranging and complex issue. The 460-page report submitted by the citizens and the profusion of legal appendices it contains (CCC, 2020) is stark evidence that expert input turned out to be significant. This was due in particular to the contribution of the legal advisory group, a body with no precedent in other citizens’ assemblies, as previously noted. Was expert input so significant as to diminish the citizens’ creativity and ultimately their role as primary contributors to their proposals? To answer this question, we examine the experts’ contributions in providing background, support and feedback to the citizens.

The background knowledge was provided by external speakers invited by the governance committee. We noted a lack of structure in the way technical information was conveyed to the citizens. The criteria that motivated who would be invited as an expert were not systematically made explicit and academic expertise and advocacy were not systematically differentiated. On several occasions, citizens made specific invitation requests that were not followed up on. When several experts were invited to speak on a specific topic, they were typically given turns to articulate their views, but no opportunity to challenge each other’s evidence.

Support with drawing up the proposals was provided by the experts from the technical and legal advisory groups. The interactions between experts and citizens were sustained in all thematic groups—not without friction. We witnessed situations in which experts went beyond their role, either unduly pushing for certain measures or discarding others. In some cases, some citizens would complain, and sometimes the facilitators would intervene to make sure the citizens’ views prevailed, but this was not systematic. Moreover, in contrast to most other citizens’ assemblies (OECD, 2020), the steering bodies did not observe strict neutrality. We witnessed for instance one of the governance gommittee’s co-chairs intervene as an expert and some members of the governance committee and a guarantor give their own opinions to the citizens on some measures.

**Fig. 3:**
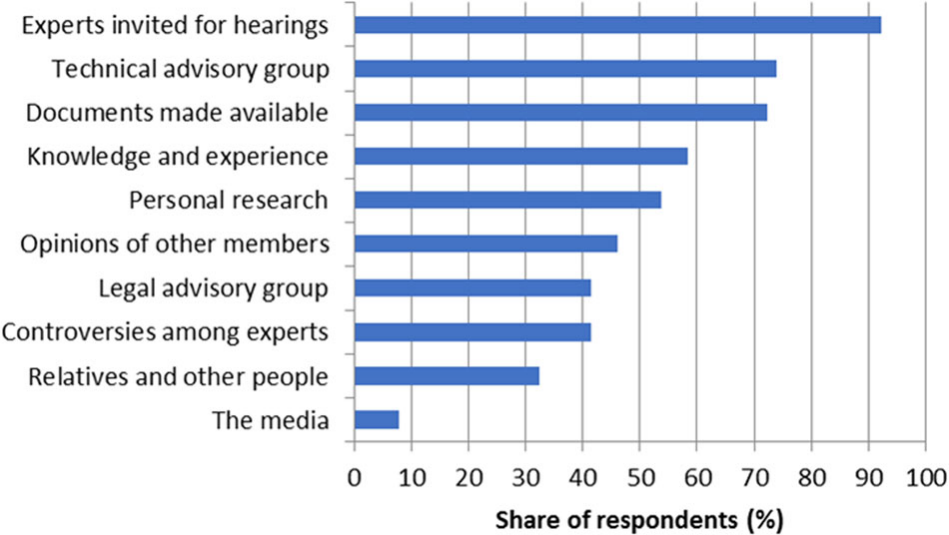
Most important sources from which the citizens formed their opinions. Surveyed in Session 7, 65 respondents.

Against these shortcomings, we observed strong demand from citizens for experts’ input and a sincere gratitude towards them. The citizens reacted strongly to the introductory presentations on climate change by Valérie Masson-Delmotte, co-chair of Working Group I of the Intergovernmental Panel on Climate Change (IPCC), many of them publicly expressing in different media how radically it had changed their attitude towards climate change. When asked in Session 7 about the most important sources from which they had formed their opinions, they most frequently mentioned the external experts (92%), the experts from the technical advisory group (74%) and the documentation made available by the organizers (72%) (65 respondents, Fig. 3). The citizens further pointed to the important role of the different steering bodies in refining and enabling their proposals, but to a limited role in proposing measures the citizens had not already thought of (63 respondents, Table 5).

**Table 5:** Survey results. Citizens’ perception of the different bodies’ roles. Session 7, 63 respondents.

	Facilitators	Experts from the legal and technical advisory group	External experts	Governance committee
They helped clarify our intentions	52	42	48	33
They respected our intentions so we could formulate them in the best possible way	52	38	22	28
They were directly involved in the formulation of intentions and objectives	21	20	13	5
They proposed measures that the citizens hadn’t thought of	10	16	27	7

Generally speaking, the citizens selected ideas that were subsequently refined into detailed proposals with the help of the legal and technical advisory groups. As a result of these sustained interactions, our view is that some measures—in particular in the building sector or in relation to the EU Common Agricultural Policy—strongly reflected expert views while others—for instance the lowering of speed limits and the regulation of the food industry—were some way removed from what most experts had suggested to the citizens. Still other measures—in particular the legal measure on the ecocide crime and the constitutional changes—were even beyond what was asked for in the engagement letter.

Lastly, but significantly, expert input did not go as far as providing feedback to the citizens about their proposals’ fitness for the 40% GHG emissions reduction target. While this might be a concern from a normative perspective, it can be argued that resources were too limited to produce such an analysis.^16^

### Decision-making

The steering bodies designed the deliberation and voting procedures framing the citizens’ decisions. Did their intervention affect the citizens’ freedom of choice?

As for deliberation, we noticed a lack of training given to the citizens in deliberative methods, which includes the prerequisites of listening to others, not interrupting, giving the floor to all, elaborating arguments and avoiding bargaining and coercion (Reber, 2016). Perhaps as a consequence, the debates were sometimes confused, with citizens interrupting one another without intervention from the facilitators. Yet at the same time, the facilitators systematically sought to avoid conflict and favored reaching consensus among citizens over organizing interim votes.^17^

We noted a similar lack of preparation with voting procedures. Voting rules were communicated to the citizens only a few hours before the sequence of votes started in Session 7. Many reacted negatively to the short notice and to the voting-by-block procedure, arguing that voting instead on a measure-by-measure basis would allow them to express their views in a more refined manner. Our view is that voting by block effectively contributed to generating high approval rates, making it more difficult for citizens to reject a specific measure without rejecting a whole block.

Despite these shortcomings, we witnessed trust build up among citizens throughout the process, partly as a result of the facilitators’ efforts. When asked in Session 7 to rate their degree of “confidence in the work of the other groups to come up with the best proposals to achieve the objectives of the Convention,” 63 respondents gave a mean grade of 7.79 (standard deviation 1.14) on a 0–10 scale. These results are in contrast with some skepticism expressed in the external survey with respect to the CCC (see Section “The general public’s perception of the citizens’ work”). This provides another explanation for the high approval rates that applied to nearly all measures, despite the fact that participants had been actively involved in drawing up only about a fifth of them—those produced by their thematic group.

### Taking stock: who is responsible for the CCC output?

The role played by each body and the citizens’ response to it is summarized in Table 6. By citizens’ assemblies’ standards, the CCC framework involved two unique bodies—the governance committee and the legal advisory group. Both provided essential inputs, the former in imposing a highly structuring framework on the proceedings, the latter in adding technical detail to the proposals to a degree well beyond that of which the citizens were capable. In contrast, the role of the guarantors’ college, the technical advisory group, the invited experts and the facilitators was more classic.

**Table 6:** Summary of interactions between the citizens and various bodies.

	Primary input	Citizens’ response
**Governance committee**	• Set the agenda, framed the thematic groups • Invited experts, mostly at its discretion • Set the voting rules, with short notice • Occasionally expressed personal views (e.g., in favor of the referendum)	• Adjusted the agenda (carbon tax, cross-cutting issues group) • Made requests for inviting certain experts (only partially acceded to) • Complained about the short notice for the voting rules
**College of guarantors**	• Oversaw the sincerity and impartiality of deliberation • Oversaw voting • Produced a report after each session • Occasionally expressed personal views (e.g., in favor of the referendum)	
**Experts invited for hearings**	• Shared their knowledge • Answered questions	• Engaged with experts • Acknowledged experts as their main source of knowledge (cf. Fig. 3)
**Technical advisory group (TAG)**	• Shared their knowledge • Gave feedback on the citizens’ proposals • Occasionally expressed personal views	• Engaged with the TAG • Acknowledged the TAG as the second most important source of knowledge (cf. Fig. 3)
**Legal advisory group**	• Reformulated the citizens’ proposals in legal terms	
**Facilitators**	• Acted as the primary entry point for the citizens • Managed deliberations	• Actively engaged with facilitators • Expressed intense gratitude to the facilitators (cf. Table 5)
**The general public**	• Contributed nearly 3400 proposals, which remained unused • Expressed skepticism towards the whole process in the external survey (cf. Section “The general public’s perception of the citizens’ work”)	• Acknowledged that they were primarily “speaking for themselves” (cf. Section “The citizens’ attitude towards the general public”) • Refused to put most of their measures to a referendum

The citizens embraced these external inputs inasmuch as it did not impair their agency, their creativity, and their freedom of choice. When they felt these values were at risk, they challenged authority. In particular, they opposed any form of hierarchical structure within their organization, as illustrated by the rejection of the cross-cutting issues group. These adjustments were made possible by flexible, sometimes even loose, planning. As a result, the CCC output can be said to have been co-produced in the sense that input from external bodies was essential, but this does not imply shared responsibility. In effect, the citizens retain full responsibility for the outptut.

## Interactions between the citizens and the general public

By design, citizens’ assemblies rely on a few selected participants to produce policy recommendations for the many. The relationship between the mini-public and the macro-public is a source of tension in the philosophy of deliberative democracy (see Section “Deliberative democracy and climate action”). The success of a citizens’ assembly depends to a large extent on the degree to which its output resonates with the will of the general public—the gold standard in this regard being when the assembly’s proposals are submitted to, and approved in, a referendum. The degree of resonance in turn depends on several features. On the one hand, the mini-public may show concern for the general public’s will and expectations. On the other hand, the general public may provide direct input to the citizens. Such mutual interest can be interpreted as a form of co-construction between the mini-public and the macro-public. In this section, we examine how the relationship between the citizens and the French population developed throughout the CCC. The findings are summarized in Table 6.

### Contributions from the general public

The CCC public website offered the possibility for anyone to make a contribution (ideas, viewpoints, proposals, etc.) to the citizens. The posts were compiled between sessions into summaries that were added to the common repository by the governance committee. Despite approximately 3400 contributions being received, this material was left largely untapped. The main reason invoked for not processing it was that the citizens were already overwhelmed with information from other sources. Given the high value the citizens seem to have attached to inputs from well-identified individuals (cf. Fig. 3), it is also likely that they dismissed inputs from anonymous, intangible sources.^18^

Besides this official channel, several organizations publicly addressed contributions to the citizens through their own website and social media. These well-identified contributions were occasionally mentioned by the citizens in the deliberation, suggesting that they had more impact than the general public’s contributions.

### The citizens’ attitude towards the general public

The citizens adopted a relatively distant attitude towards the broader public. When asked in Session 1 what best described their own role in deliberations, 36% said they would speak for themselves, 22% for themselves and on behalf of people like them, 21% for particular causes, 19% for the broader public and 3% for other groups and special interests (116 respondents).

This attitude was somewhat echoed by their handling of the referendum issue. Submitting proposals to referendum is one means of reaching out to the broader public, as illustrated by the Irish example. The CCC contained quite an original feature in this respect, the Prime Minister’s letter giving the citizens the opportunity to make the call about the referendum, although subject to the President’s endorsement. Many of the CCC’s advocates had high expectations of this provision (Pech, 2021), and prominent figures among the governance committee and the guarantors’ college more or less explicitly encouraged the citizens to push for referenda. In a striking move, however, the citizens strongly rejected (with 60–80% of the votes) that route for their technical proposals—not without having hotly debated the issue. This is yet another example of the citizens showing independence from the steering bodies. In turn, they approved the submission of constitutional reforms to a referendum. However, unlike the former technical proposals, proceeding with a referendum for constitutional reform would require the President to obtain approval from Congress. In other words, from a purely procedural perspective, the citizens agreed to request the difficult referendum, but not the “easy” ones. In the end, with the anticipated congressional disapproval of the proposed referendum, none will be organized. How did the citizens get there?

An argument commonly advanced by opponents to the referendum was that the general public would not be as “enlightened” in their voting as the members of the Convention had become. We note here that, from a normative perspective, such an argument echoes Lafont (2015)’s concern that a mini-public could be an illegitimate shortcut to the broader public (see Section “Deliberative democracy and climate action”). Another argument was that the broader public would vote for or against the President of the Republic, instead of voting for or against the Convention’s proposals. Some counter-argued that such a voting strategy could be avoided by allowing people to approve of items from a menu of options, instead of approving a single package. Yet another widely shared motive for not supporting the referendum was the anxiety many citizens expressed at the prospect of having to campaign for the Convention’s measures in the public debate, were a referendum to be held. Getting involved in this way was resented as being well beyond what they had consented to when they agreed to participate in the CCC. To sum up, the citizens were torn between some sort of empowerment, some feeling that their measures were too good to be left for others to decide, and anxiety, others having difficulties endorsing measures beyond the framework of the CCC. Interestingly, though apparently diametrically opposite, these tensions contributed to the same outcome: discarding referenda.

This is not to say, however, that the citizens had no concern at all for the general public. In the deliberation, some repeatedly emphasized that the citizens’ proposals should be politically “credible.”^19^ This was especially the case in the plenary debate about the two most sensitive proposals—the reduction in working hours, eventually rejected, and the reduction of speed limits on motorways, eventually adopted by a small majority—many among their opponents arguing that adopting these measures would undermine the CCC’s credibility with the general public.

### The general public’s perception of the citizens’ work

The external survey generated two important, and somewhat contradictory, insights (Fabre et al., 2021). On the one hand, support for the CCC’s proposals was found to be broad among the population and stable across the two waves. Specifically, when asked to approve of the measures proposed for referendum, the respondents in the external survey expressed strong majority support (i.e., beyond 65%) for all measures (Fabre et al., 2021, Fig. 12). On the other hand, despite lacking awareness of the CCC (22% knew about it in Wave 1, 42% in Wave 2), the respondents expressed strong skepticism about the representativeness of the pool of citizens (74% among CCC-aware respondents in W2) and about the government’s handling of the process, deemed “useless as the government [would] only take on the measures it likes” and “a government communication operation.” They expressed more mixed feelings when surveyed about citizens’ assemblies in general, only a minority showing confidence in “the ability of randomly chosen citizens to deliberate productively on complex issues” (32% in W1, 28% in W2) while a majority supported the creation of a permanent “assembly made up of 150 randomly chosen citizens, with a right of veto on the texts voted in Parliament” (68% in W1, 63% in W2).

### Taking stock: co-construction without the general public?

In the light of these facts, our view is that the CCC developed as an autonomous body whose primary goal was to produce a consistent set of measures. Such internal consistency was seen as a way to be as transformative as the target implied and to resolve the inherent tension between significant GHG emissions reductions and the social justice imperative. To achieve this, the citizens made an intensive yet selective use of the inputs provided by experts and technical advisors. In contrast, they somewhat ignored comments from the general public, which they resented as too specific, thus missing the significant effort they were putting into making the measures coherent with one another. This attitude somewhat isolated the citizens from the general public.

Another result of this attitude, the citizen’s handling of the referendum may appear to be a missed opportunity. The external survey suggests that, had the citizens seized that opportunity, the broader public would have approved of their measures. This is however subject to two important caveats. First, as subsequent experience showed, nothing guarantees that the President would not have applied a “filter” of some sort to their recommendations. Second, nothing guarantees that support among the population would have remained high until the referendum, especially not with the high level of skepticism that emerged among the population, legitimizing fears of insincere voting.

## Taking stock: researching the CCC

Our research protocol was unique in terms of its size—it actively involved thirty academics, instead of a handful in other contexts (the Irish assemblies and CAUK, for instance)—and comprehensiveness—it combined qualitative observations and repeated surveys, both internal and external. Such an ambitious setup allowed us to produce insights which we hope will be useful to both academics and policy-makers. This was achieved in a rather bottom-up way, with small teams coordinating with one another without strong centralization. It did not come without difficulty, however, and important lessons can be drawn.

First, while most plenary discussions were recorded by the organizers, this was not the case for the myriad group and table discussions. Despite significant efforts to cover as many of those as possible, it was technically impossible for us to follow everything. In any case, we had to resort to our own audio equipment, which unfortunately produced too many hours of wasteful recordings, leaving us with pretty much only our written notes to exploit. Recording and transcribing the proceedings as systematically and professionally as possible would greatly enhance research possibilities.

Second, the research charter we adhered to (see Supplementary Appendix A) was meant to avoid interference with the process—the so-called Hawthorne effect. This goal was by and large fulfilled, save for one unintentional breach. In the Session 2 outburst that is described in Section “Agenda-setting”, one citizen pointed to a question from our questionnaire as proof of a hidden agenda aiming to get the citizens to endorse the carbon tax. In fact, our internal survey had not been subjected to a pilot test and, after reconsidering the questionnaire, we recognized that a few questions could be interpreted as biased. We immediately proceeded with reformulating them in a more neutral way. This was a sobering reminder that pilot tests are essential, and accordingly should be planned well ahead.

Third, the governance committee granted us very full access to the proceedings and we could effectively follow pretty much everything we wanted to. That said, we felt our presence was more tolerated than welcomed by the governance committee representatives and, to a lesser extent, by the facilitators. Strikingly, we were never given a chance to officially introduce ourselves, our research interests and our protocol, to the citizens.^20^ This is unfortunate, for we think such an introduction would have helped avoid the misunderstanding that caused the Session 2 outburst. This feeling that we had from the outset only got worse after that incident. Likewise, we could only rely on ourselves for the distribution of the questionnaire and the collection of the filled forms. Unlike in other assemblies (CAUK in particular), the citizens received very little encouragement to fill in our questionnaires. Despite short meetings between the researchers and the representatives from the governance committee at the beginning of each session, the situation did not significantly improve over the course of the CCC. In the end, however, this somewhat adverse context only marginally impaired the tremendous research possibilities offered to us by the organizers.

To conclude, a very full access to the proceedings, a research force of about twenty permanent academics and adherence to a principled charter were not sufficient to prevent significant losses (in terms of recordings and responses to the internal survey) nor some interference with the process. To facilitate research and increase its effectiveness, we recommend sustained dialog between researchers and organizers at every stage of the process. The former should pay the fullest attention to drawing up and testing their protocol and the latter should embrace the researchers’ work and brief the citizens about its potential benefits for society at large.

Our group has now entered a post-assembly phase freed from the restrictions placed in the charter. Some of us are conducting in-depth interviews with key stakeholders, including voluntary citizens and members of the steering bodies. This will provide original material for further research.

## Conclusion

The CCC was the central part of a 3-year sequence of political events that shook up France’s climate policy, from the Gilets Jaunes crisis to the Grand National Debate to a new law adapting some of the CCC’s proposals. Its proceedings were seriously disrupted by national labor unrest and a global pandemic. In such an adverse context, the largest experiment to date with climate assemblies produced an outcome containing no fewer than 149 measures, some of which could be game-changing for France’s climate policy. While the CCC’s work can be said to have created political momentum, owing to the intense parliamentary activity it generated, follow-up is actually quite limited—to the citizens’ disappointment. In the light of its specific characteristics, what can we learn from this experience?

The CCC was intended to follow an “innovative approach to co-construction of solutions.” This elliptical injunction implied that some external actors would play an active role. While this may be justified by the complexity inherent in climate issues, it is somewhat at odds with the primacy given to randomly chosen individuals in a citizens’ assembly. In this paper, we focus on the interactions developed by CCC participants with various partners for co-construction and ask: Was external input so significant as to undermine the citizens’ responsibility for the final output? Did the citizens’ work resonate with the broader public?

Regarding the first question, we provide evidence that the steering bodies imposed significant framing effects—e.g., in the type of expertize the citizens were exposed to—yet kept the framework flexible enough to be responsive to the citizens’ concerns—allowing them for instance to adjust the agenda, in particular by dismissing the carbon tax issue. The framework proved effective at building consensus among the citizens and trust with respect to the steering bodies, to the point of creating a strange situation in which the citizens express ex post satisfaction with the process, but not with the outcome.

Regarding the second question, we found that the relationship the citizens developed with the general public was one of mutual skepticism. To some extent the citizens worked in isolation from the general public in an effort to keep their proposals consistent and coherent. This attitude culminated in them not submitting their proposals to referendum, despite being given the opportunity to do so.

Ultimately, the “co-constructive” approach to the CCC succeeded in the narrow sense of bringing the citizens and policy-makers closer together, but not in the broader sense of bringing the citizens closer to the broader public. What lessons can be drawn from the deliberative democracy theory perspective? First, the comprehensiveness of the proposals and the wide consensus that surrounded them confirm that citizens’ assemblies can contribute to improving the quality of deliberation. An important qualification here is that this was achieved with significant help from experts—which is somewhat normal with climate issues—and organizers—which is more peculiar. In contrast, the outcome gives little support to the claim that citizens’ assemblies serve as a “body of trustees” to the broader public. This is due to a missed rendez-vous: the public showed mixed feelings about the process—support for the citizens’ proposals, skepticism about how the government would follow up—while the latter denied the former the chance to participate in a referendum. Partly as a result of this, the CCC provides yet another piece of evidence of the lack of consequentiality in citizens’ assemblies, despite having generated significant parliamentary activity. Lastly, the opportunity given to the citizens to make the call about the referendum—a provision that is, to our knowledge, unique to the CCC—served as a grand experiment, generating interesting insights. On the one hand, the citizens’ refusal illustrates that concerns that a citizens’ assembly could be a shortcut to the general public are not unfounded. On the other hand, it is somewhat proof by contradiction that pairing a referendum and a citizens’ assembly can be a way to improve both consequentiality and legitimacy.

With climate assemblies mushrooming at the sub-national, national (e.g., in Germany, Spain, Scotland, Denmark, Finland, and Austria), European and international levels and citizens’ assemblies being applied to a range of new issues (including vaccination and genome editing), what then should be emulated from the French CCC, and what should not? Items to be emulated include the responsiveness of the framework that helped empower the citizens and keep them engaged even after deliberation was over. While we view this as a positive outcome, we note that it raises a yet unspoken issue as to the extent to which individual citizens should be kept in the loop in the post-assembly phase.

As for aspects to be avoided, the French CCC lacked a clear commitment structure, such that, despite promoting co-construction, it did not trigger political uptake. The issue took a dramatic turn with the intense personification of the CCC, framed as a “no filter” interaction between the President of the Republic and the citizens. The “no filter” commitment generated widespread comment and, whatever it meant, it can be said to have been defaulted on by the President on at least two occasions—first by claiming “veto cards”, second by having the government re-work the citizens’ proposals before submitting them to Parliament. Ironically, the outcome could have been different had the citizens submitted their proposals to referendum. Our view here is that a more transparent and outspoken commitment structure as to how the government might respond to the citizens’ proposals could make the citizens’ assembly’s mandate more straightforward to both the participating citizens and the general public.

## Supplementary information

Appendices

## Data Availability

The observational data generated during the CCC and analyzed in the current study are included in the published article. The individual questionnaires and descriptive statistics from the internal survey can be found here: https://www.participation-et-democratie.fr/donnees-de-recherche-sur-la-convention-citoyenne-pour-le-climat. The questionnaire for the external survey can be found here: https://preferences-pol.fr/doc_q.php#_c. More detail can be found in Section “Materials and methods”. The official material can be found at: https://www.conventioncitoyennepourleclimat.fr/.

## Footnotes

1 The second most significant example is the United Kingdom’s Climate Assembly (CAUK). Ireland also held a climate assembly in 2016 (Devaney et al., 2020). For an account of ongoing climate assemblies, see www.knoca.eu.

2 The related concepts of co-production and co-creation are similarly regarded as a “catch-phrase” (Brandsen et al., 2018), characterized by “fuzziness, an innate goodness (who can be against any of it?) combined with a supple application to diverse phenomena” (Brandsen and Honingh, 2018).

3 The nuances mainly have to do with the stage considered. Contrasting co-construction and co-production, Vaillancourt (2009) associates the former with the design stage of public policy and the latter with the implementation stage. Contrasting co-creation and co-production, Brandsen and Honingh (2018) associate the former with the initiation and planning stages and the latter with the design and implementation stages. Building on these categorizations, we propose the following articulation between the three concepts: co-creation upstream, co-production downstream, and co-construction in the middle (i.e., at the core design stage).

4 While co-construction and its parent concepts are generally framed as a bilateral interaction, the involvement of professionals and market actors leads some to view it rather as tripolar, i.e., a set of interactions between the state, the market and civil society (Vaillancourt, 2009).

5 This is also known as the input-throughput-output framework (Caluwaerts and Reuchamp, 2015; Courant, 2020).

6 Adaptation to climate change was not mentioned in the letter. It was effectively left outside the scope of the proceedings.

7 The CESE represents civil society in the third Assembly of the Republic, alongside the National Assembly and the Senate. It is a Constitutional Assembly which advises the Executive on legislation. Its members include non-governmental organizations, unions, business representatives and students.

8 In December 2020, EU member states agreed to tighten this target to 55%.

9 Protection of the environment was deemed important with an average score of 8.95 (on a 0–10 scale) among CCC participants, versus 7.87 in the population.

10 The transcript is available in French at https://www.elysee.fr/front/pdf/elysee-module-15714-fr.pdf

11 Despite claiming to play only three “veto cards,” the President effectively rejected more measures. While the citizens proposed to ban domestic flights when a train alternative of less than four hours was available, he lowered this threshold to two and a half hours. He also rejected organizing a referendum on recognition of the crime of ecocide, a measure he nevertheless committed to re-work with the government and back at European level.

12 The citizens recommended that the following part be added: “The Republic guarantees the preservation of biodiversity and the environment and fights against climate change.”

13 https://www.legifrance.gouv.fr/jorf/id/JORFTEXT000043956924

14 The Congress of the French Parliament (*Congrès du Parlement français*) is the name given to the body created when both houses of the present-day French Parliament—the National Assembly and the Senate—meet at the Palace of Versailles to vote on revisions to the Constitution or to listen to an address by the President of the French Republic (Source: Wikipedia).

15 The issue was deemed settled by the governance committee, considering that nuclear power already significantly contributes to France’s relatively low GHG emissions.

16 By citizens’ assemblies standards, the budget of the CCC was already quite substantial—€6.7 million, to be compared with the £500,000 allocated to its closest counterpart, the CAUK. To get a sense of the extra cost needed for impact assessment, consider that it typically takes France several government officials working full-time for several months to assess the country’s pledges to the Paris Agreement.

17 On rare occasions, citizens’ opinions were surveyed by show of hands, which does not preserve anonymity and thus threatens sincere voting.

18 One could also expect the output from the Grand National Debate—a much wider consultation involving 1.9 million contributions—to provide the starting point for the CCC, which was supposed to be continuation of it. As a matter of fact, these contributions were ignored in the CCC.

19 Credibility was also invoked in the financial sense that each proposal should be matched by adequate funding.

20 The only communication was an eight-minute video footage of the researchers’ work shot in Session 3 and published online soon after: https://www.youtube.com/watch?v=tQAyvVjExPM
