# Supplementary material for: “Co-construction” in deliberative democracy: lessons from the French Citizens’ Convention for Climate
Source: Humanit Soc Sci Commun. 2022 Jun 22;9(1):207. doi: 10.1057/s41599-022-01212-6 (PMC9214676; doi:10.1057/s41599-022-01212-6)
Supplement: Supplementary file 1 — Appendices [file 41599_2022_1212_MOESM1_ESM.pdf]

Humanities & Social Sciences Communications |https://doi.org/10.1057/s41599-022-01212-6

SUPPLEMENTARY MATERIAL

"Co-construction" in Deliberative Democracy: Lessons from the French Citizens' Convention for Climate

Louis-Gaëtan Giraudet[1], Bénédicte Apouey, Hazem Arab, Simon Baeckelandt, Philippe Bégout, Nicolas Berghmans, Nathalie Blanc, Jean-Yves Boulin, Eric Buge, Dimitri Courant, Amy Dahan, Adrien Fabre, Jean-Michel Fourniau, Maxime Gaborit, Laurence Granchamp, Hélène Guillemot, Laurent Jeanpierre, Hélène Landemore, Jean-François Laslier, Antonin Macé, Claire Mellier, Sylvain Mounier, Théophile Pénigaud, Ana Póvoas, Christiane Rafidinarivo, Bernard Reber, Romane Rozencwajg, Philippe Stamenkovic, Selma Tilikete, Solène Tournus.

Content

Appendix A: Observation charter 2

Appendix B: Presidential announcement of the CCC 4

Appendix C: Prime Minister's engagement letter 5

[1] Contact for correspondence : giraudet@centre-cired.fr

Appendix A: Observation charter

The charter was translated with www.DeepL.com/Translator, with only minor edits by the authors.

The Citizens' Convention for Climate is an unprecedented event in French democratic life and a crucial moment for the orientation of climate policies. The Governance Committee wishes to facilitate access to research teams who want to make direct observations of its proceedings and produce data useful for various research works. The resulting observation, analysis and critique will be valuable in order to draw lessons for the future from this original exercise in deliberative democracy.

At the invitation of the Governance Committee, various French and foreign researchers and doctoral students, as well as participation practitioners have expressed their interest in observing the deliberations of the Citizens' Convention and studying its proceedings for research purposes. The Committee will be careful to coordinate the planned work and will ensure the diversity and plurality of approaches proposed by the researchers. To this end, the Governance Committee deems it necessary to come up with a single common questionnaire proposed to the participants. It wishes to define by mutual agreement a framework for the presence of observers that does not disrupt the smooth running of the Citizens' Convention and, above all, the work of the citizens.

The Governance Committee asks the observers to respect the following rules and guidance for the relations they will establish during their observations of the Citizens' Convention.

In order to fully understand how the Citizens' Convention will unfold and the facilitation protocol that will be used, the Governance Committee invites them to attend a presentation with the facilitators that will take place on Friday, October 4 at 11:00 a.m. at the CESE.

Relations and exchanges with the Governance Committee and the facilitators

Observers are requested to identify themselves to the Convention's chief facilitators at its first working session on Friday, 4 October, upon arrival in the Convention room.

At the opening of each session of the Citizens' Convention, the main moderator will inform the participants of the presence of observers (role and nature), except in those moments when a closed session seems necessary for the smooth running of the session.

The total number of observers who may take part in a session is limited to one person per table during group work, i.e., 20 people in plenary session.

Relations and exchanges with participants during the work of the Citizens' Convention tables

In order not to disrupt the work of the citizens during the table deliberations, only one observer will be allowed to attend their discussions.

He or she will have to introduce him or herself to the table participants and indicate whether he or she wishes to make an audio recording of the proceedings. The participants are free to refuse him or her access or the recording of their comments.

Above all, he or she must respect the dynamics of each table, without interfering in any way in the exchanges between the participants during all working hours. His or her presence will remain discreet and mute.

He or she will have to adopt a neutral stance in all his or her exchanges with the participants with regard to the Citizens' Convention and the issues being discussed, so as not to influence them.

During breaks or lunch, observers will be able to freely exchange with participants, maintaining a neutral stance and taking care not to take them away from the group for more than a few minutes, for which informal collective moments are important. For this reason, observers will not be able to participate in dinners with citizens.

Questionnaires to participants and personal data

In order not to overly solicit citizens, only one anonymous questionnaire may be submitted to participants at the beginning and one at the end of each session of the Citizens' Convention. Each questionnaire should not exceed 15 minutes in order to fit easily into the planned facilitation process. The wish to submit these questionnaires will be communicated to the participants at the opening of each session. The facilitators will encourage them to answer them on a voluntary basis. Before each session of the Citizens' Convention, the questionnaires to be submitted at the beginning and end of the session will be sent to the Governance Committee for information. The database containing the questionnaires at the end of the Citizens' Convention will be made available to all research teams wishing to analyse them.

Personal data may be requested from participants during the last session of the Citizens' Convention, for the purpose of interviews to be conducted with consenting persons after the end of the Convention. The collection of this data can only be done with the person's written and informed consent. Observers will forward the planned informed consent form to the Governance Committee prior to its release. The database collecting these personal data will be kept by the CESE and may be made available to teams who justify the need for it for research purposes.

Relations and exchanges with the media

Observers may respond to the media if questioned. However, the Governance Committee asks them not to do so during the sessions of the Citizens' Convention, so as not to disrupt the work of the Convention. It also asks them to maintain a neutral stance throughout the Citizens' Convention with regard to its proceedings and the issues addressed. The Governance Committee reserves the right to exclude any observer who does not comply with the rules set out in this charter, or whose behaviour in any way disrupts the proceedings of the Convention.

Appendix B: Presidential announcement of the CCC

This is an extract of the press conference President Macron gave on April 25th, 2019 in closing the Grand National Debate. The text is fully available at: https://www.elysee.fr/emmanuel-macron/2019/04/25/conference-de-presse-grand-debat-national. The extract was primarily translated with www.DeepL.com/Translator, with minor by the authors. We emphasize the elements that are discussed in the paper.

The first of these transitions, the most urgent, the most imperative, is obviously the climate. The climate must be at the heart of the national and European project. The climate emergency is here, our youth are telling us so at every moment and our fellow citizens want to act. They are already taking action on a daily basis, they want us to help them go further, to accompany them, to help them find concrete solutions, but there is a citizen's awareness of these issues that has been profoundly transformed in recent years and is moving much faster than many public policies. So a lot has been done in the last two years, I can come back to this when answering your questions. Next week we will go further in terms of energy policy and in the coming weeks in terms of circular economy to fight against all forms of waste. But I want us to be able to change our method more strongly to respond more concretely and radically to expectations. **Changing the method means first of all using more collective intelligence on this subject. We have many solutions, I have often said, but they are often too complex for our citizens, not used, not well known, not well adapted, whether it is the help to change the boiler, to change the vehicle, it is improving but finally there is much to do. This is why the first mission of the citizens' convention, 150 citizens drawn by lot in June, will be to work on this subject, to redesign all the concrete measures of aid to citizens on the climate transition in the field of transport, housing renovation (whether insulation or heating) to make them more efficient, to define if necessary other incentives or constraints and, if necessary, to define additional resources and propose funding to do so. What comes out of this convention, I pledge, will be submitted without filter either to a vote in parliament or to a referendum or to direct regulatory application.** And then the second change in method is that I want us to set up an ecological defense council that will bring together the Prime Minister, the main ministers in charge of this transition, and the major State operators, which I will chair on a regular basis in order to both make strategic choices and put this climate emergency at the heart of all our policies, and to ensure that it is followed up in all ministerial changes when a direction is taken. Finally, the success of this transition will be ensured in all ministerial changes when a direction is taken. Finally, the success of this transition depends on our European ambition, i.e. our ability to defend a minimum carbon price at the European level, a carbon tax at the borders and a more ambitious green finance. I can also come back to this if you have any questions.

Appendix C: Prime Minister's engagement letter

The engagement letter was addressed by Prime Minister Edouard Philippe to Head of CESE Patrick Bernasconi on July 2nd, 2019. It is reproduced in full here. The translation primarily used www.DeepL.com/Translator, with minor edits added by the authors. We emphasize some of the key elements discussed in the paper.

Mr. President,

The yellow vests crisis, the success of the Grand National Debate, the numerous signatories of the petition known as the "Case of the century," the mobilization of the youth, demonstrate the desire of many of our fellow citizens to participate more closely in the development of public policies, starting with environmental policies.

On April 25, the President of the Republic announced the creation of a citizens' convention to respond to the dual demand for more participation and more ecology expressed by the French. Its purpose is to involve the whole society in the ecological transition, through a representative sample of citizens, and to mobilize collective intelligence to move from consensus on the diagnosis to compromise on solutions, and to initiate a profound transformation of our lifestyles. In addition to the High Council for the Climate and the Ecological Defense Council, it represents a change in method and governance to accelerate the ecological transition.

The convention will be composed of 150 citizens chosen by lot and representative of the diversity of society. Its mandate will be to define structuring measures to achieve, in a spirit of social justice, a reduction of greenhouse gas emissions of at least 40% by 2030 compared to 1990. At the end of its work, the Convention will publicly submit a report to the Government and the President of the Republic on its discussions, as well as all of the legislative and regulatory measures that it deems necessary to achieve the objective of reducing greenhouse gas emissions. **It may designate, among the legislative measures, those it deems appropriate to submit to a referendum.** The government will respond publicly to the proposals made by the Citizens' Convention and will publish a provisional timetable for the implementation of these proposals. **The Convention will be able, if necessary and if it wishes, to express an opinion on the government's responses.**

I would like the Economic, Social and Environmental Council to organize the work of this citizens' convention by setting up a governance committee bringing together the Ministry of Ecological Transition and Solidarity, personalities qualified in the field of ecology, participatory democracy and economic and social issues, and representatives of the Citizens' Convention who will be appointed later.

This committee will have autonomy of decision in the accomplishment of its missions which will be the following: to ensure the steering of the convention, to support it in setting up the agenda, to supervise its implementation, to define its rules of procedure and its working methods. Finally, technical and legal support will be provided to ensure the legal transcription of the proposals.

In order to allow the organisation of the first meeting of the Citizens' Convention by mid-September at the latest, the governance committee will have to define the modalities of the drawing of lots and all the points necessary for its launch by mid-July at the latest.

To guarantee the independence of the Convention, **a college of guarantors will also be appointed: it will ensure that the work of the Convention is carried out in accordance with the principles of impartiality and sincerity**. I propose that you, the President of the Senate and the President of the Economic, Social and Environmental Council each appoint a guarantor.

This innovative procedure for co-construction of solutions is a process to which the President of the Republic attaches a determining importance in order to accelerate the ecological transition, which is a priority of the governmental action. I know that I can count on your involvement and that of the whole of organized civil society represented within the EESC, which you chair, to carry out this important mission.

Please accept, Mr. President, the assurance of my best wishes.
